# Enhanced Intestinal Permeation of Doxorubicin Using Chitosan Nanoparticles

**DOI:** 10.15171/apb.2018.048

**Published:** 2018-08-29

**Authors:** Marziyeh Zare, Soliman Mohammadi Samani, Zahra Sobhani

**Affiliations:** ^1^Faculty of Pharmacy, Shiraz University of Medical Sciences, Shiraz, Iran.; ^2^Department of Quality Control, Faculty of Pharmacy, Shiraz University of Medical Sciences, Shiraz, Iran.

**Keywords:** Doxorubicin, Chitosan nanoparticles, Oral delivery, Intestinal permeation, Everted rat gut method

## Abstract

***Purpose:*** Due to limited oral bioavailability of doxorubicin (Dox) many efforts during the last decades focused on the development of novel delivery systems to overcome these limitations. In the present study, Dox encapsulated chitosan nanoparticles were prepared to evaluate the intestinal permeation of Dox via oral administration.

***Methods:*** Nanoparticles were fabricated based on ionic gelation method using tripolyphosphate. Some physicochemical properties, such as nanoparticle size and morphology, loading efficiency and in vitro drug release in 3 different pH values (5.0, 6.8 & 7.4) were evaluated. Intestinal permeations of free Dox and Dox loaded in nanoparticles were assessed using rat intestinal sac model.

***Results:*** The nanoparticles were spherical shape with average size of 150 ± 10 nm. The entrapment and loading efficiency of Dox were up to 40% and 23%, respectively. According to the release profiles, up to 30% of loaded drug was released within 6hrs and the remaining amount of Dox was released more gradually, but this pattern was related to pH of the medium. The amount of drug released at acidic condition (pH 5.0) was greater than other pHs. The intestinal permeation of Dox increased nearly up to 90% by loading in chitosan nanoparticles.

***Conclusion:*** Using chitosan nanoparticles presents a potential safe drug delivery system for oral administration of Dox. In vivo studies and the determined pharmacokinetic and pharmacodynamic of Dox loaded chitosan nanoparticles after oral administration are planned for future studies.

## Introduction

Oral administration of drugs is one of the most common approaches. It has many advantages in comparison with intravenous rout, such as better safety, ease of administration, lower cost, convenience and patient compliance.^1-3^ The common regimen for chemotherapy consists of the administration of anticancer drugs through i.v. injection or infusion. The poor physicochemical and biopharmaceutical properties of various anticancer drugs and biological barriers in the GI tract restrict the oral administration of these drugs.^4^ Utilizing pharmaceutical approaches, such as nanocarriers can improve the oral absorption of such drugs.^3^

Polymeric nanoparticles, particularly chitosan nanoparticles have special features that can improve the oral absorption of drugs. Chitosan is the polycationic derivative of chitin with mucopolysaccharide structure similar to cellulose.^5-9^ Chitosan nanoparticles have many advantages over natural polymers. The preparation methods of chitosan nanoparticles are simple and mild, and the resulting particles have good stability, and low toxicity (LD50>16g/kg). This nanoparticles can be used for different purposes. Hence, they are being considered as a drug delivery carrier, especially in recent years. Chitosan has a linear structure with a number of free amine groups, and can easily form ionic cross-linkage with multivalent anions.^10^ The mucoadhesive properties of chitosan increase the contact time of a drug with the absorption site, leading to increased absorption. This property is based on the interaction between the positive charge of the chitosan and the negative charge of the mucin in the intestine.^11^ Furthermore, many studies have shown that chitosan can affect the tight junctions and induce their temporary opening, which increases the permeation of drugs through the intestinal epithelium.^3,10-13^ The effect of chitosan and its derivatives on the oral absorption of some anticancer drugs has been studied.^10,12^

Dox is an anticancer drug with excellent properties, which is being used to treat several malignancies, such as lymphoma, osteosarcoma and other sarcomas, carcinomas, and melanoma.^14-17^ Dox is an anthracycline glycoside antibiotic that inhibits the DNA synthesis and cell proliferation. Dox is one of the essential drugs in cytotoxic and adjuvant medicine of 20^th^ WHO essential medicine list (EML) for adult and 6^th^ for children. It is administered through IV infusion. However, there is the possibility of unwanted side effects due to high initial concentration or its toxicity followed by fast decay if its administered below the minimum therapeutic level.^13,18^ A relatively longer systemic exposure time with less fluctuation, can lead to lower toxicity and improved efficacy, which can be provided by oral delivery of this drug.^13,18^

During this project chitosan nanoparticles were prepared by ionic gelation method and the effect of some parameters, such as polymer concentration, poly anionic agent concentration, mixing speed, etc. on particle size were analyzed. Dox was loaded in the selected nanoparticles, and the intestinal permeability studies were carried out by intestinal sac model.

## Materials and Methods

Low-molecular weight chitosan and pentasodium tripolyphosphate (TPP) were purchased from a domestic supplier of Sigma-Aldrich (USA) in Iran. Doxorubicin hydrocloride was obtained as a 2mg/ml solution in 0.9% (w/v) sodium chloride from EBEWE Pharma (Austria). All other chemicals, solvents and reagents used were of chemical or analytical grade as needed, which were used without further purification. All aqueous solutions were prepared with deionized water.

### Preparation of Dox loaded chitosan nanoparticles

Chitosan nanoparticles loaded Dox (Dox-Cs-NPs) were prepared using an ionic gelation method. Nanoparticles were formed spontanously, by dropwise adding of TPP aqueous solution to the chitosan aqueous solution (in 1% v/v acetic acid) under stirring at room temperature. Probe sonicator or magnet stirrer were used to mix chitosan and TPP solutions. The effect of chitosan and TPP solutions concentration, and different mixing methods on the particle size and size distribution were analyzed. Selected formulations with the particle size less than 300 nm ^3^ and narrow size distribution were used for loading experiments. Dox was added into chitosan solution at different drug: polymer ratio (w:w) prior to adding TPP solution. The suspension was centrifuged at 15000 rpm for 30 min, and sedimented nanoparticles were washed with deionized water and lyophilized before storage.

### Characterization of NPs

#### Particle size and particle shape analysis

Nanoparticle sizes were determined using a laser diffraction particle size analyzer (Microtrac, Nano-flex 180°, USA) at room temperature.

The shape and surface characteristics of the nanoparticles were observed by scanning electron microscopy (SEM) (TESCAN vega3,Czech).

#### Drug loading and encapsulation efficiency

The encapsulation efficiency of Dox-Cs-NPs was determined by indirect method. Dox-Cs-NPs were separated from the suspension by ultracentrifugation (Hettich, Model Mikro220R, Germany) at 15000 rpm and 4°C for 30 min. The amount of free Dox in the supernatant was analyzed by HPLC.^19-22^ The encapsulation efficiency (EE) was calculated by equation 1:

(eq. 1) EE%= ((T−F)/T)*100

And drug loading amounts (LA) were determined using equation 2:

(eq. 2) LA%= ((T−F)/W)*100

Where F is the free amount of drug in the supernatant, T is the total amount of drug added into the chitosan solution, and W is the weight of Dox-Cs-NPs. All formulations were carried out in triplicate.

The chromatographic system for Dox analysis consist of a Hichrom C18 column (250 × 4 mm, UK) and a Cecil instrument (Adept series and CE 4200 UV/Vis Detector, England). The mobile phase consisted of acetonitrile: distilled water (30:70 v/v) and the pH of the mobile phase adjusted to 3 using phosphoric acid. The flow rate was set to 1 mL/min and Dox was quantified at 233 nm. The HPLC method validation showed the linearity of method (r^2^ = 0.9993) in the range of 1–50 µg/ mL. CV% was less than 9% and the accuracy was more than 88% within this range of concentrations (1–50 µg/ mL). The specificity was tested in the presence of the nanoparticles components.

#### In vitro drug release study 

Profile of Dox release from the nanoparticles was analyzed in three different pH values of 5.0, 6.8 and 7.4 in phosphate buffer solution. Dox-Cs-NPs were suspended in 0.5 mL buffer solution in the microtubes. The tubes were put into shaker incubator (BIOER Mixing Block MB-102, China) and temperature was set to 37°C. At scheduled time points, the nanoparticles were separated by centrifugation at 10000 rpm for 10 min, and the released drug in the supernatant was measured by HPLC method as described earlier. Following supernatant decantation, the same volume of fresh pre-warmed buffer solution was replaced. All measurements were performed in triplicate. The release percentage at each time was calculated and cumulative released curves are depicted.

#### Analysis of release data

To describe the drug release mechanism, release data were analyzed using the Ritger and Peppas equation:

(eq. 3) $$M_{t}/M_{\infty }=Kt^{n}$$

Where M_t_ represents the amount of drug released in time t, M_∞_ is the total amount of drug that should be released at infinite time, K is constant and “n” is the release exponent indicating the type of drug release mechanism.^23^

To assay the release kinetic, data were evaluated by zero order, first order, and Higuchi model. For zero order release kinetic, the cumulative percent of released drug versus time was assessed. In the cases of first order kinetic and Higuchi model, the logarithm of the amounts of the remaining drug to be released versus time, and the cumulative percent of released drug versus square root of time were evaluated, respectively. The relevant correlation coefficients were used to determine the best model.

#### Intestinal permeation studies by intestinal sac model

#### Preparation of everted intestinal sac

Permeation of free Dox and Dox loaded chitosan nanoparticles across the intestinal wall was studied. Everted and normal intestinal sacs were prepared according to the standard procedure.^24-26^

Male Sprague Dawley rats (250-300g) were used for the intestinal permeation experiments. Animals were sacrificed and a longitudinal segment as long as 30 cm of the upper section of intestine was removed and rinsed with cold oxygenated physiological Ringer solution and cut into segments of 2 cm length. Each sac tied to a cotton thread and filled with 2 mL Ringer solution. For everting the intestine segment a glass rod was carefully used and the serosal side of the intestine was filled with 0.5 mL Ringer solution (acceptor part). Sacs were placed in a jacket glass tubes containing 40 mL physiologic Ringer solution that were continually bubbled with 95% O_2_ and 5% CO_2_ kept at 37°C (donor chamber).

#### Intestinal permeation 

After equilibration at 37°C for 5 min, the exact volume of Dox solution or nanosuspension of Dox-Cs-NPs were added to the donor chamber. At scheduled time points after adding the drug to the donor chamber five intestinal sacs were removed. The liquid inside the sacs were centrifuged and analyzed with HPLC method. In the case of adding Dox-Cs-NPs to the donor chamber, the liquid inside the sacs at first was treated with acetic acid 1% and then ultrasonicated for 45 min to dissolve chitosan and to extract the entrapped drug within the nanoparticles. Finally, the solution was centrifuged and analyzed with HPLC.

## Results and Discussion

### Preparation of Dox-Cs-NPs

Preparation of chitosan nanoparticles was performed by using various concentrations of chitosan (0.2-0.5mg/mL) and TPP solutions (0.2-0.5mg/mL).^27^ Chitosan nanoparticles were prepared through ionic gelation technique. This method is based on electrostatic interaction between the positive charge of amine groups of chitosan and negative charge groups of polyanion, such as tripolyphosphate. This technique is simple and performed in the aqueous medium. By adding TPP to the chitosan solution, nanoparticles were spontaneously formed during stirring at room temperature. For mixing the nanosuspension the effects of magnetic stirring and ultrasonic wave on the particle size and size distribution were analyzed. The results of particle size are shown in Table 1. Dispersion of the NPs with probe sonicator was much better than magnetic stirrer, and the homogenisity of the particle size improved when probe sonicator was used.

**Table 1:** Particle size of nanoparticles obtained by two methods of mixing. Data were reported as mean (n=3)

**Formulation** **No**	**Chitosan Conc** **(W/V%)**	**TPP Conc (W/V%)**	**Nanoparticle Mean Size (nm)- Number size**	**Nanoparticle Mean Size (nm)- Volume size**	**Mixing Methods**
F_1_	0.2	0.2	52.4	418	**Magnet Stirrer**
F_2_	0.3	0.2	49.3	572	
F_3_	0.5	0.2	228.2	285.3	
F_4_	0.2	0.3	2449	2521	
F_5_	0.3	0.3	2582	2691	
F_6_	0.5	0.3	39.7	503	
F_7_	0.2	0.5	1765	1822	
F_8_	0.3	0.5	6000	6000	
F_9_	0.5	0.5	3470	3520	
F_10_	0.5	0.1	440	738	**Probe Sonicator**
F_11_	0.5	0.25	418	781	
F_12_	**0.5**	**0.3**	**152.675**	**341.75**	
F_13_	0.5	0.5	Unread	-	

**Figure 1:**
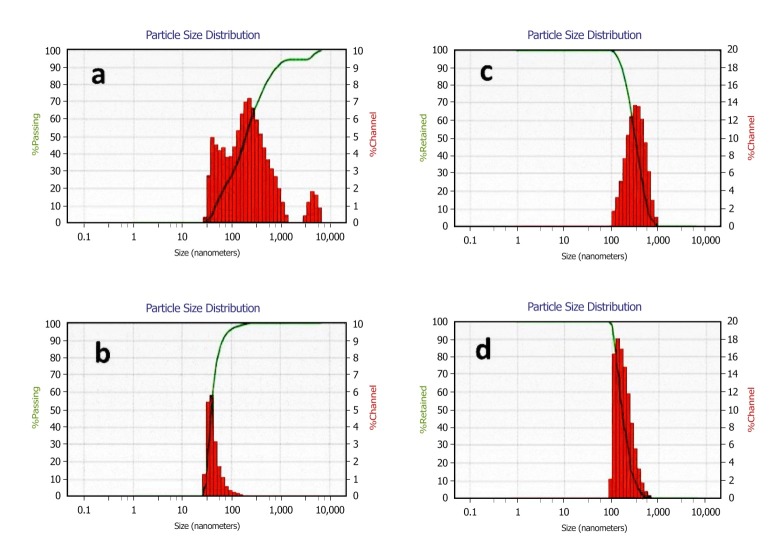
Volume (top) and number (bottom) size and size distribution of nanoparticle obtained by magnetic stirrer (a,b) and probe sonicator (c,d)

The poly dispersity of obtained NPs with two methods is shown in Figure 1. The selected formulation had the minimum size (volume diameter 152.6 nm) and was prepared by adding 2 mL of TPP (0.3%w/v) to 3 mL of chitosan solution (0.5%w/v) and sonicated for 120 sec by 75% amplitude. This formulation was selected for Dox loading. Dox was added into chitosan solution at different drug: polymer ratio (w:w) prior to the addition of TPP solution.

**Figure 2:**
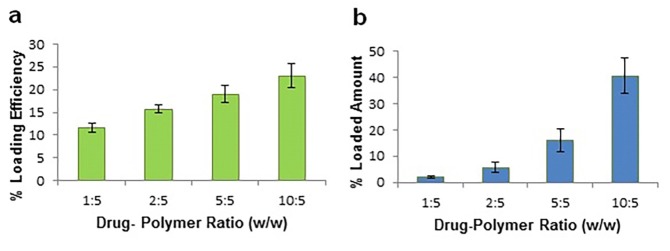
Loading efficiency (a) and loaded amount (b) of Dox in chitosan nanoparticles. Data were reported as mean ± SD (n=3)

Drug loading and encapsulation efficiency is shown in Figure 2. Dox were successfully entrapped into chitosan nanoparticles with the encapsulation efficiency up to 23% and loading amount of 40%.

### Characterization of Preparation of Dox

#### Morphology studies

**Figure 3:**
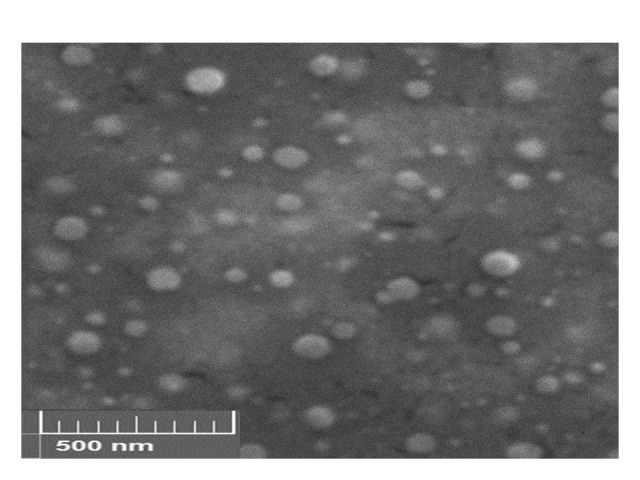
Scanning electron microscopic image of Dox-Cs-NPs

The morphology of Dox-CS-NPs was assessed by SEM and the results are shown in Figure 3. As it can be seen, the particles are spherical with smooth surface. No aggregation was detected and the particle size in the micrographs confirms the results of laser diffraction particle size analysis.

#### In vitro drug release

**Figure 4:**
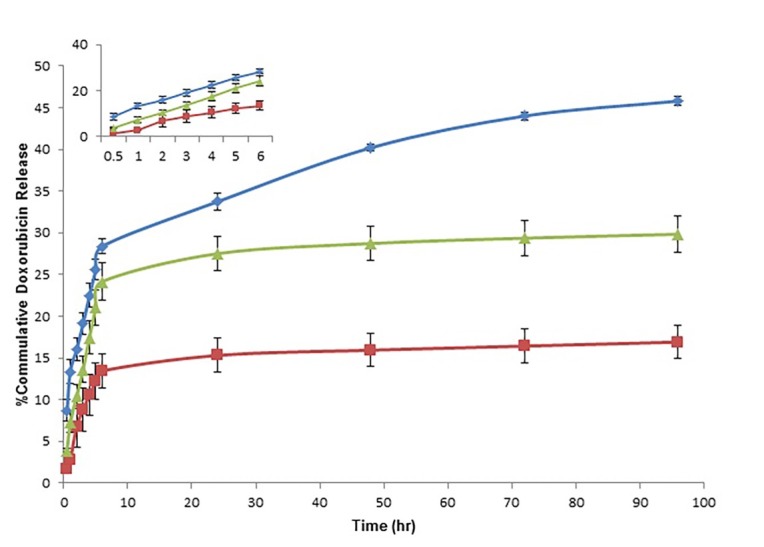
In vitro release profile of Dox from Dox-Cs-NPs in three different pH values. Blue line is related to pH 5, green to pH 7.4 and red to pH 6.8

The release profile of Dox from Dox-Cs-NPs at three different pH values is shown in Figure 4. A quick release was seen from Dox-Cs-NPs in the first 6 hrs (approximately 30% of the drug was released at pH 5.0) and a relatively slow and sustained release was observed in the following days. By decreasing the pH from 7.4 to 5.0, the percentage of the released drug increased, but by changing the pH of the release medium to 6.8 the amount of drug released was reduced. The amount of drug released at acidic condition (pH 5.0) was greater than other pHs. This phenomenon is suitable for drug release at tumor region.^28^ Another positive aspect of these nanoparticles is the release profile at pH 6.8. At this pH, the lowest amount of drug was released. Since in the *in vivo* condition the range of intestine pH is similar to this point, the Dox-Cs-NPs will have slight release before absorption. These differences in the release behavior at three pH media could be explained as the influence of protonation of amine groups of Dox, chitosan and changing the solubility of drug and polymer in these conditions.^28^

According to the Figure 4 the release of Dox from chitosan nanoparicles occurred in 2 different manners. At first, a fast release profile was observable showing that some part of Dox was absorbed on the surface of the nanoparticles and this part was released at the beginning up to the first 6 hrs showing a burst release, and the second part of the drug could be released up to 96 hrs from the beginning. In this regards release kinetics were assessed in two different time intervals. Release kinetic was evaluated for 0-6 hrs and 6-96 hrs, separately. Release kinetic was calculated by data fitting to zero order, first order, Higuchi model, and Ritger and Peppas equation. The relevant correlation coefficients were used to determine the best model (Table 2). According to the results, the drug release followed the Higuchi model. The mechanism of drug release is highly related to the pH. It seems that Fickian diffusion at pH 5.0 and anomalous mechanism (Fickian diffusion and polymer relaxation) at pH 7.4 and 6.8 are the major drug release mechanisms. In addition, erosion of the chitosan nanoparticles at pH 6.8 is also involved.

#### Intestinal permeation

**Figure 5:**
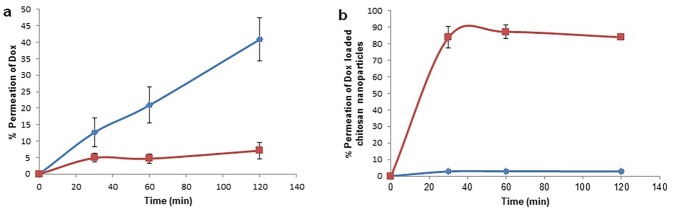
Intestinal permeation of Dox solution (a) and Dox loaded in chitosan nanoparticles (b) across the everted (red line) and not- everted (blue line) rat gut

To evaluate the role of chitosan nanoparticles in the intestinal permeation of Dox, everted rat gut sac technique was employed. This is a simple and accurate model.^24,26,27^ Permeation of Dox from simple solution and Dox-Cs-NPs across the everted and not- everted gut is shown in Figure 5. The transport of Dox encapsulated in the nanoparticles was significantly higher than the free Dox (approximately12.7 folds). When Dox solution was placed at the mucosal surface of intestine, the very low amount of drug (7%) transported across the intestinal wall (Figure 5a). In spite of the proper log P of Dox (log P=1.3) for diffusion across the intestinal wall, presence of P-glycoprotein pumps (P-gp pumps) as an efflux pumps in the luminal part of the enterocytes could show this discrepancy. In contrast, Dox can diffuse from the serosal site to mucosal site of intestine. Assessment of the Dox loaded Cs-NPs permeation via everted gut and bypassing the P-gp pump showed that permeation of the drug will be increased profoundly up to 89% (Figure 5b). The presence of chitosan nanoparticles increased the intestinal permeation of Dox approximately by 12.7 folds. This observation also showed that when Dox is entrapped into the chitosan nanoparticles, it is not available for P-gp pumps and can be transported across the intestinal wall. In addition, this significant increase in the intestinal permeation could be explained by the effect of chitosan on the opening of tight junctions and facilitation of the paracellular transport of Dox. However, in the not everted gut experiment, simple diffusion of drug was inhibited by entrapping the drug into the nanoparticles. Therefore, no Dox could be transport from serosal to mucosal site.

**Table 2:** Results of release kinetics according to various equations

**R** ^ 2 ^ ** Correlation of Doxorubicin Chitosan Nanoparticles Release Data**								
**Release Model**	**Zero Order**		**First Order**		**Higuchi**		n*	
**pH**	**0-6 (hr)**	**6-96 (hr)**	**0-6 (hr)**	**6-96 (hr)**	**0-6 (hr)**	**6-96 (hr)**	**0-6 (hr)**	**6-96 (hr)**
5	0.9804	0.9476	0.9846	0.9374	0.9882	0.9885	0.4393	0.1793
6.8	0.935	0.7891	0.9641	0.7893	0.9806	0.9051	0.9127	0.07
7.4	0.9957	0.7492	0.9971	0.7563	0.9846	0.88	0.7074	0.0707

n*: Pepas mechanism slope equation line

## Conclusion

By using the chitosan nanoparticles, the intestinal permeation of Dox approximately reached to 90%. Considering this effect, chitosan nanoparticles can present a promising platform for the development of oral delivery of poorly absorbable drugs. *In vivo* studies and determination the pharmacokinetic and pharmacodynamic of Dox loaded chitosan nanoparticles after oral administration are planned for future studies.

## Acknowledgments

This project was part of Pharm. D thesis of the Marziyeh Zare. The authors would like to thank Shiraz University of Medical Sciences, Vice Chancellor of Research for their financial support (grant No 11114). They also wish to thank Mr. H. Argasi at the Research Consultation Center (RCC) of Shiraz University of Medical Sciences for his invaluable assistance in editing this manuscript.

## Ethical Issues

The protocol of the animal study was approved by the local Ethics Committee of Shiraz University of Medical Sciences (approval number 11114).

## Conflict of Interest

Authors state that there is no conflict of interest.
